# Are there differences between unconditional and conditional demand estimates? implications for future research and policy

**DOI:** 10.1186/1478-7547-6-15

**Published:** 2008-08-05

**Authors:** Budi Hidayat

**Affiliations:** 1 Department of Health Policy and Administration, Faculty of Public Health, University of Indonesia, Indonesia

## Abstract

**Background:**

Estimations of the demand for healthcare often rely on estimating the conditional probabilities of being ill. Such estimate poses several problems due to sample selectivity problems and an under-reporting of the incidence of illness. This study examines the effects of health insurance on healthcare demand in Indonesia, using samples that are both unconditional and conditional on being ill, and comparing the results.

**Methods:**

The demand for outpatient care in three alternative providers was modeled using a multinomial logit regression for samples unconditional on being ill (*N *= 16485) and conditional on being ill (*N *= 5055). The ill sample was constructed from two measures of health status – activity of daily living impairments and severity of illness – derived from the second round of panel data from the Indonesian Family Life Survey. The recycling prediction method was used to predict the distribution of utilization rates based on having health insurance and income status, while holding all other variables constant.

**Results:**

Both unconditional and conditional estimates yield similar results in terms of the direction of the most covariates. The magnitude effects of insurance on healthcare demand are about 7.5% (public providers) and 20% (private providers) higher for unconditional estimates than for conditional ones. Further, exogenous variables in the former estimates explain a higher variation of the model than that in the latter ones. Findings confirm that health insurance has a positive impact on the demand for healthcare, with the highest effect found among the lowest income group.

**Conclusion:**

Conditional estimates do not suffer from statistical selection bias. Such estimates produce smaller demand effects for health insurance than unconditional ones do. Whether to rely on conditional or unconditional demand estimates depends on the purpose of study in question. Findings also demonstrate that health insurance programs significantly improve access to healthcare services, supporting the development of national health insurance programs to address under-utilization of formal healthcare in Indonesia.

## Background

Several published studies on healthcare demand estimate the probabilities of using healthcare services conditional on being ill sample [1-4]. The ill sample is usually generated from self-assessments of health status. Conditional estimates are the preferred method because an individual's decision to seek treatment implies that they are ill, which is especially true in developing countries. Estimations of healthcare demand, therefore, often rely on estimating these marginal and conditional probabilities.

However, estimating healthcare demand conditional on the event of illness poses several problems. First, there may be an association between self-assessed health status and healthcare use [5], raising the possibility of endogeneity (on the grounds that there are unobservable factors correlated with both the likelihood to report illness and to seek health care). The estimated responses of health care demand to exogenous variables based on an ill sample only would therefore be biased [6]. Second, conditional estimates may also be susceptible to an underreporting of the incidence of illness in surveys and, hence, would yield only a lower-bound estimate [7]. Finally, the total effects of prices on the demand can be inferred only from unconditionalestimation [8] and such estimations would produce *long-run *price effects [6].

This study examines the effects of health insurance on the demand for outpatient care, using the second round of the Indonesian Family Life Survey. The analysis was based both on samples of unconditional responses and on samples of responses conditional on being ill. To construct the latter sample, this study used a definition of sickness that more accurately identifies people more likely to have used healthcare services. Individuals included in the definition were those who reported having at least one activity of daily living (ADL) impairment and/or a serious illness. This approach identified 5055 individuals in the conditional sample, around 31% of the total unconditional sample (N = 16485).

The purpose of this study is two-fold: first, to compare the results of two approaches estimations – unconditional and conditional estimates; second, to investigate the effects of health insurance on the use of public and private outpatient care.

The setting for this study is the country of Indonesia. Located in Southeast Asia, Indonesia is an archipelago consisting of more than 17,000 islands. With a population of 231.6 million in 2007, Indonesia is the fourth largest country in the world after China, India and the United States [9]. Inadequate access to formal health care is a serious problem in Indonesia. Following the economic crisis during 1997–1998, the proportion of household survey respondents who reported an illness or injury and sought care from a modern health care provider declined by 25% [10]. A policy option to improve access to formal health care has been articulated by enacted the National Social Security Law (UU No. 40/2004), which is now used as a basis for introducing a national health insurance program.

This article contributes more evidence on the relative magnitudes of conditional and unconditional demand effects on healthcare demand. It also adds to the existing evidence base by analyzing the effect of health insurance programs on healthcare demand in the context of a developing country. In particular, this article provides evidence on whether proposing a national health insurance program would be welfare-enhancing in terms of increasing access to formal healthcare in Indonesia.

## Methods

### Data – Indonesian Family Life Survey

This study uses data from the second round of the Indonesian Family Life Survey (IFLS2), a panel survey carried out by the RAND Corporation in conjunction with Indonesian researchers and various international agencies. The first round of survey (IFLS1) included interviews with 7,224 households covering 22,347 individuals within those households. The second round of the survey, IFLS2, re-contacted the same households interviewed in IFLS1 and successfully re-interviewed 6,751 (93.5%) of the IFLS1 households. An overview of the IFLS1 and IFLS2 survey is described elsewhere [11,12].

### Estimation – Multinomial Logit

The demand for healthcare is a function of health insurance and a set of exogenous variables. The dependent variable is outpatient care during the previous four weeks of interview in three provider options: self-treatment, public and private. I estimated a multinomial logit (MNL) model in the form [13]:

(1) $$\begin{array}{ccc} \mathrm{Pr}(Y_{i}=j)=\frac{e^{{\beta^{\prime }}_{j}{\text{x}}_{i}}}{\sum_{k=0}^{2}e^{{\beta^{\prime }}_{k}{\text{x}}_{i}}}, & \text{for} & j=0,1\text{ or }2 \end{array}$$

Equation (1) was estimated using the maximum likelihood procedure. The reference group is those who used self-treatment. The vector x_*i *_represents a set of exogenous variables and *β *represents regression parameters to be estimated. The estimated equations above provide a set of probabilities for the *j+*1 choices for an individual with characteristics x_*i*_.

The MNL model assumes that the stochastic portions of the conditional utility functions are uncorrelated across alternatives. The model therefore requires the assumption of 'independence of irrelevant alternatives (IIA)' be satisfied [13]. To validate this assumption, both a Hausman specification and Small-Hsiao tests of IIA assumption were employed. Another alternative to the MNL, which is based on a reasonable distributional assumption on the behavior of the disturbance term, is a nested multinomial logit (NMNL). Yip et al. (1998) pointed out that the NMNL model produces essentially the same results as the MNL model [14].

To ascertain the pure effects of insurance, specifically on changes in the predicted probability of insurance across income groups and to show the magnitude effects implied by the coefficients, I used the recycling prediction method [15]. From the MNL estimation, the predicted probabilities were calculated by changing only insurance status and income quintile, while holding all other characteristics of the sample constant.

Table 1 provides a complete list of the variables used, with their definitions and descriptive statistics. The exogenous variables (*x*_*i*_) that were used in the analysis are detailed below.

**Table 1:** Definition variables used in the analysis

**Exogenous** ** variable**	**Definition**	**Unconditional**		**Conditional**	
		
		Mean	SDev	Mean	S.Dev
Askes	1 if govt-employ insurance; 0 otherwise	0.098	0.298	0.101	0.301
Jamsostek	1 if priv-employ insurance; 0 otherwise	0.052	0.222	0.047	0.213
Askes*Inc.	Interaction *Askes *and income	0.162	0.752	0.166	0.801
Jamsostek*Inc.	Interaction *Jamsostek *and income	0.071	0.409	0.066	0.392
Symptoms	1 if had ≥ 1 symptom; 0 otherwise	0.797	0.402	0.879	0.327
ADLs limit	1 if had ≥ 1 limited ADL; 0 otherwise	0.244	0.429	0.795	0.404
Vgood GHS^R^	Very good health status	0.090	0.286	0.067	0.249
Good GHS	General health status was good	0.798	0.401	0.707	0.455
Poor GHS	General health was bad & very bad	0.112	0.315	0.226	0.418
Serious ill	1 if had serious ill; 0 otherwise	0.113	0.316	0.367	0.482
Female	1 if female; 0 otherwise	0.551	0.497	0.731	0.444
HHs size	Number of household members	5.852	2.554	5.987	2.693
Married	1 if married; 0 otherwise	0.836	0.370	0.874	0.332
No-schooling^R^	Had no education	0.121	0.326	0.167	0.373
Elementary	Had some primary education	0.472	0.499	0.467	0.499
Junior	Had some secondary education	0.136	0.342	0.124	0.329
Senior	Had some senior education	0.201	0.401	0.176	0.381
High	Had some higher education	0.070	0.256	0.066	0.249
Age (years)	Individual age in years	36.64	11.55	39.69	12.46
Ln. income	Log natural per-capita income (Rp)	11.080	0.856	11.126	0.867
Electricity	1 if had electricity; 0 otherwise	0.867	0.340	0.871	0.335
Ln. travel-cost	Log one way travel-costs to health post	9.765	8.981	10.194	8.852
Ln. travel-time	Log one way travel-time to health post	15.040	3.143	14.965	3.110
Urban	1 if urban; 0 otherwise	0.480	0.500	0.501	0.500
Region: Jakarta^R^	Jakarta residence	0.092	0.289	0.107	0.309
Sumatra	Lived in Sumatra	0.199	0.399	0.217	0.412
West Java	Lived in West Java	0.171	0.376	0.183	0.387
Central Java	Lived in Central Java	0.186	0.389	0.141	0.349
East Java	Lived in East Java	0.141	0.348	0.091	0.287
Bali & WNT	Lived in Bali and WNT	0.110	0.313	0.150	0.357
Kalimantan	Lived in Kalimantan	0.045	0.207	0.056	0.230
Sulawesi	Lived in Sulawesi	0.055	0.228	0.055	0.229

Sample size (*N*)		16,485		5,055	

^R^Indicate the reference (omitted groups) in the MNL regressions.

#### Health Insurance

Health insurance is expected to improve demand for healthcare. Two types of health insurance programs were included in the model: (i) health insurance for government employees, known as *Asuransi Kesehatan *(*Askes*) and (ii) health insurance for private sector employees, known as *Jaminan Sosial Tenaga Kerja *(*Jamsostek*). The *Askes *represents a mandatory insurance that covers all civil servants, pensioners of civil servants and armed forces. It also covers their families and survivors. The scheme provides the benefit of comprehensive health care, provided mainly through public health facilities. The *Jamsostek *scheme covers private employees and their dependents up to a maximum of three children. Benefits include comprehensive health services through both public and private providers [16].

Health insurance programs in this study are assumed to be exogenous given that such programs are mandated either by the government or employers, and hence unobservable individual factors to join particular health insurance scheme are not likely to be a serious problem. If insurance is indeed endogenous, then evaluating the impact of insurance on healthcare demand without correcting for endogeneity will yield biased estimates [17-19]. To guarantee that health insurance is indeed exogenous, I tested for the possible endogeneity of insurance using the following two steps [17]. First, a reduced form of insurance participation was estimated using a probit model (a first-stage regression). This regression included all covariates in the demand equation in addition to proposed identifying variables. Second, the predicted values of the insurance variable derived from the first-stage regression and the observed values of the insurance variable were then included in the demand equation. If the predicted coefficient for insurance is not significant, then one can assume that health insurance is an exogenous variable. Testing for endogeneity was also performed using an instrumental variable (IV) estimation [20].

#### Health

Three measures of individual health status were taken into account: symptoms, activity of daily living (ADL) impairment, and general assessment of health status (GHS). Individuals who reported having at least one symptom and one difficulty of ADL impairment were grouped as having symptoms and ADL impairment, respectively. GHS respondents were reclassified into three groups: very good, good and poor (aggregated from very bad and bad of the GHS). A dummy variable indicating whether an individual had a serious illness in the last four years was also included. The severity of the disease was self-reported.

Since the study used a sample that was conditional on being ill, health status was also potentially endogenous due to a sample selection problem [5,6]. A probit model with the sample selection was carried out to investigate whether conditional estimates are affected by selection bias [21,22].

#### Income

Income is considered an important determinant of the demand for healthcare. This study used household expenditure as a proxy for income. Information about income is biased and difficult to assess in many developing countries, particularly in subsistence farming households. Income data is also typically prone to under-reporting and measurement error, ignoring the contribution of own production and in-kind transfers. Household expenditures were adjusted with the 1997 consumer price index data, using Jakarta as a reference in order to correct for price differences in various locations. To control the effect of household size, per-capita household expenditures were used. For the remainder of the paper, expenditures are referred to as income.

The effects of insurance may differ across income groups. An interaction term for insurance and income was therefore included in the model. This interaction allows one to test whether income has different effects of insurance on the demand.

Other variables that were considered and included are: female (1/0), household size, married (1/0), education (a dummy variable indicating: no school [the reference] elementary, junior, senior and high), electricity (1/0), age (years), one way travel cost (Rupiah) and travel time (minutes) to health facilities, and urban (1/0). To control for regional differences, dummy variables for the regional location of the survey site were also included.

## Results

**Figure 1:**
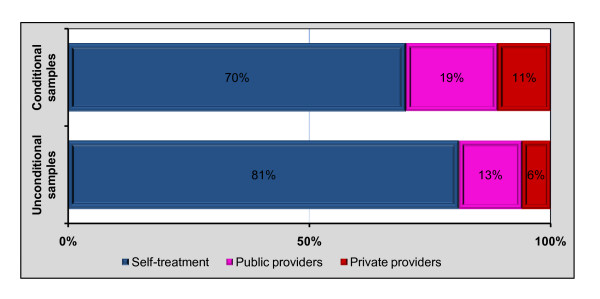
The distribution of providers used four-weeks prior to the IFLS survey.

Figure 1 shows that 70% of ill individuals used self-treatment, 19% saw a private provider and the remaining 11% sought a public provider. The distribution of unconditional samples was 81%, 13%, and 6% for self-treatment, private and public provider, respectively.

### Testing the Endogeneity of Insurance

Results of the endogeneity test suggest that having health insurance is indeed an exogenous variable (i.e., the predicted value of the insurance variable when inserted in the demand equation is not significantly different from zero). The predicted value of insurance was generated from a probit model of insurance participation. This was estimated separately for *Askes *and *Jamsostek*, using identifying variables and all other exogenous variables in the MNL model. The identifying variables used included: employment status of the household head (whether public or private employee); whether individual were active in community meetings or water organizations, and; whether an individual's relationship to the household head is as a spouse. These variables were selected as appropriate instruments since they turned out to be insignificant in the demand equation, but were highly correlated with insurance participation. *R*^2 ^for the insurance equation (first-stage regression) in the unconditional estimate was 0.31 and 0.21 for *Askes *and *Jamsostek*, respectively. While for conditional estimate, it was 0.26 and 0.31 for *Askes *and *Jamsostek*, respectively.

The validity of the instruments was also tested using an over-identification restrictions test, i.e., Sargan-test statistic [13,20]. The test did not reject the null hypothesis that the instruments were uncorrelated with the error term of the demand function in all cases. In unconditional estimates, the *p*-values of the Sargan-test for the public and private models were 0.36 and 0.11, respectively. Whilst in conditional estimates, the *p-values *were 0.513 and 0.363 for the public and private models, respectively. This suggests that the models are reasonably well specified and the instruments are valid.

Using the IV estimation, the endogeneity test also failed to reject the null hypothesis. Table 2 reports summary statistics for testing the endogeneity of health insurance derived from the IV estimation. The test for both *Askes *and *Jamsostek *in all cases was not significantly different from zero, indicating that the suspected endogenous variable is indeed exogenous, and no corrections for endogeneity are needed.

**Table 2:** Summary statistics testing for the endogeneity of the health insurance variable

Statistics Tests*	**Public providers**			**Private providers**		
		
	DF**	Statistic	*p*-value	DF**	Statistic	*p*-value
**Unconditional estimates (N = 16485):**						
*Askes & Jamsostek*						
-Wu-Hausman	F(2,16453)	0.7326	0.4807	F(2,16453)	0.19261	0.8248
-Durbin-Wu-Hausman	Chi-sq(2)	1.4679	0.4800	Chi-sq(2)	0.38597	0.8245

*Askes *only						
-Wu-Hausman	F(1,16454	0.0850	0.7707	F(1,16454)	0.34298	0.5581
-Durbin-Wu-Hausman	Chi-sq(1)	0.0851	0.7705	Chi-sq(1)	0.34361	0.5578

*Jamsostek *only						
-Wu-Hausman	F(1,16454)	0.8811	0.3479	F(1,16454)	0.18927	0.6635
-Durbin-Wu-Hausman	Chi-sq(1)	0.8828	0.3475	Chi-sq(1)	0.18962	0.6632

**Conditional estimates (N = 5055):**						
*Askes & Jamsostek*						
-Wu-Hausman	F(2,5023)	0.14599	0.8642	F(2,5023)	1.34468	0.2607
-Durbin-Wu-Hausman	Chi-sq(2)	0.29383	0.8634	Chi-sq(2)	2.70505	0.2586

*Askes *only						
-Wu-Hausman	F(1,5024)	0.00437	0.9473	F(1,5024)	0.47523	0.4906
-Durbin-Wu-Hausman	Chi-sq(1)	0.00439	0.9472	Chi-sq(1)	0.47811	0.4893

*Jamsostek *only						
-Wu-Hausman	F(1,5024)	0.24074	0.6237	F(1,5024)	2.64705	0.1038
-Durbin-Wu-Hausman	Chi-sq(1)	0.24221	0.6226	Chi-sq(1)	2.66198	0.1028

*Statistic tests were calculated using Instrumental Variable estimation [15].

** Degree of freedom (DF).

### Sample Selection Model

As noted earlier, conditional estimates are likely to be biased. A probit model with a sample selection was employed using the 'heckprob' command in STATA [15]. Determinants of sickness included all covariates that were used in the demand equation plus several other indentifying variables. The instruments used included: smoking status; household head's employment status (whether public or private employee); whether individuals used a septic tank for defecation; whether individual were involved in community activities, and; four dummy variables indicating type of garbage disposal (e.g. collected, burned, discarded on premises, and other). The results of the probit model with a sample selection yielded an insignificant correlation between the error terms – i.e., Chi-squared(1) = 0.02, with a *p*-value 0.88 – ruling out any possibility of sample selection bias [22].

### Model Estimation Results

Table 3 displays the results of unconditional (left panel) and conditional (right panel) demand estimates. The last row of the table reports R-squared values as well as the results of IIA assumption tests. The R-squared values suggest that the covariates explain 14% and 12% variation in the unconditional and conditional models, respectively. Both Hausman and Small-Hsiao tests indicated that the MNL model passed the IIA assumption, suggesting that retaining the present model does not lead to inconsistent estimates [13].

**Table 3:** MNL estimation results using self-treatment as the comparison group

	**Unconditional estimates**				**Conditional estimates**			
		
	Public Providers		Private providers		Public providers		Private providers	
		
	*β*^a^	[se]^b^	*β*^a^	[se]^b^	*β*^a^	[se]^b^	*β*^a^	[se]^b^
Askes	0.654^‡^	[0.101]	0.125	[0.141]	0.511^‡^	[0.153]	0.023	[0.193]
Jamsostek	0.512*	[0.270]	1.362^‡^	[0.187]	0.314	[0.377]	1.086^‡^	[0.344]
Askes*Inc	0.065*	[0.040]	-0.014	[0.049]	-0.031	[0.067]	0.019	[0.053]
Jamsostek*Inc.	-0.760^‡^	[0.239]	-0.388^‡^	[0.112]	-0.529*	[0.286]	-0.599^‡^	[0.228]
Symptoms	1.955^‡^	[0.123]	2.436^‡^	[0.220]	1.287^‡^	[0.176]	2.704^‡^	[0.454]
ADLs limit	0.257^‡^	[0.059]	0.390^‡^	[0.079]	0.233*	[0.132]	0.373^‡^	[0.142]
Vgood GHS^R^								
Good GHS	0.359^‡^	[0.114]	0.472^‡^	[0.148]	0.372*	[0.196]	0.396*	[0.238]
Poor GHS	1.383^‡^	[0.126]	1.698^‡^	[0.164]	1.421^‡^	[0.207]	1.645^‡^	[0.251]
Serious-ill	0.537^‡^	[0.073]	0.847^‡^	[0.084]	0.491^‡^	[0.103]	0.859^‡^	[0.126]
Female	0.604^‡^	[0.056]	0.250^‡^	[0.074]	0.548^‡^	[0.100]	0.371^‡^	[0.124]
HHs size	0.007	[0.011]	0.048^‡^	[0.013]	0.003	[0.016]	0.023	[0.019]
Married	0.644^‡^	[0.100]	-0.198*	[0.102]	0.744^‡^	[0.169]	-0.021	[0.160]
No-schooling^R^								
Elementary	0.089	[0.080]	0.372^‡^	[0.141]	0.074	[0.113]	0.424^†^	[0.177]
Junior	0.038	[0.108]	0.459^‡^	[0.168]	0.024	[0.164]	0.400*	[0.228]
Senior	0.039	[0.108]	0.512^‡^	[0.164]	0.000	[0.164]	0.606^‡^	[0.219]
High	-0.343^†^	[0.151]	0.714^‡^	[0.185]	-0.08	[0.221]	0.875^‡^	[0.259]
Age (years)	-0.001	[0.003]	0.004	[0.004]	-0.001	[0.004]	-0.004	[0.005]
Ln. income	0.069*	[0.039]	0.431^‡^	[0.051]	0.038	[0.059]	0.380^‡^	[0.077]
Electricity	0.495^‡^	[0.083]	1.144^‡^	[0.198]	0.368^‡^	[0.126]	0.919^‡^	[0.248]
Ln. travel-cost	0.026^‡^	[0.009]	0.027^†^	[0.012]	0.024*	[0.013]	0.055^‡^	[0.018]
Ln. travel-time	0.004	[0.003]	0.003	[0.004]	0.001	[0.005]	-0.002	[0.006]
Urban	-0.384^‡^	[0.061]	0.193^†^	[0.084]	-0.377^‡^	[0.092]	-0.002	[0.124]
Region:Jakarta^R^								
Sumatra	0.324^‡^	[0.119]	-0.264^†^	[0.127]	0.05	[0.172]	-0.575^‡^	[0.185]
West Java	0.314^‡^	[0.118]	-0.053	[0.117]	0.280*	[0.170]	0.129	[0.164]
Central Java	0.242^†^	[0.121]	0.163	[0.122]	0.183	[0.180]	-0.082	[0.188]
East Java	0.516^‡^	[0.130]	0.578^‡^	[0.137]	0.365*	[0.200]	0.552^‡^	[0.206]
Bali & WNT	0.825^‡^	[0.127]	0.301^†^	[0.144]	0.483^‡^	[0.180]	0.149	[0.196]
Kalimantan	0.692^‡^	[0.149]	-0.902^‡^	[0.256]	0.576^‡^	[0.211]	-1.261^‡^	[0.368]
Sulawesi	0.604^‡^	[0.151]	-0.490^†^	[0.236]	0.441^†^	[0.219]	-0.649*	[0.338]
Constant	-7.121^‡^	[0.504]	-13.031^‡^	[0.686]	-5.766^‡^	[0.793]	-12.365^‡^	[1.081]

*N*	16,485				5055			
Pseudo *R*^2^	0.144				0.118			
Wald Chi-sq(58)	2308.17; sig. 0.000				782.78; sig. 0.000			

Hausman test	16.7 (omitted-public), *p*-val = 0.98				1.18 (omitted-public), *p*-val = 1.00			
IIA: *X*^2 ^(30)	13.1 (omitted-private), *p*-val = 0.99				5.11 (omitted-private), *p*-val = 1.00			

Small-Hsiao	41.7 (omitted-public), *p*-val = 0.08;				18.3 (omitted-public), *p*-val = 0.95			
test IIA: *X*^2^(30)	16.5 (omitted-private), *p*-val = 0.98				34.1 (omitted-private), *p*-val = 0.28			

^a^The estimated parameters *β*; superscript ‡,†, and *significance at 1%, 5%, and 10% level, respectively.

^b^Robust standard errors given in [brackets].

^R^References (omitted groups).

The MNL estimates show that the coefficient estimate for *Askes *insurance was positive for public and private providers, but only significant for the former with a *p*-value at the 1% level. The findings hold true for both unconditional and conditional estimates. The coefficient estimate of the interaction between *Askes *and income resulted only in a positive and significant effect for public services providers for the unconditional sample. The effect was negative for the conditional sample but not statistically significant.

The coefficient estimate of *Jamsostek *insurance in the unconditional estimates was positive for both provider types, although there was a difference in the level of significance (i.e., 10% at public providers and 1% at private ones). While in the conditional estimates, the coefficient of *Jamsostek *was significant for the private provider only. The coefficient estimate of the interaction (between *Jamsostek *and income) was negative for both provider types and significant at the 1% levels, except for public provider in the conditional estimates. The negative coefficients of the interaction terms taken together suggest that the effects of *Jamsostek *insurance on the probability of using formal health care were higher among the poor.

Results of most covariates were consistent with expectations. A general picture emerges that both unconditional and conditional estimates yielded similar results with respect to the direction of most covariates. This includes health status, gender, household size, marital status, education, income, electricity usage and travel costs.

### Recycling Prediction Results

This section presents the results of the recycling prediction method to ascertain the pure effects of insurance and to show the magnitude effects implied by the coefficients. Based upon unconditional and conditional MNL estimations, I predicted the probabilities of using outpatient care (self-treatment, care with public providers and care with private providers) by changing only the health insurance status while holding all other variables at their mean. Three scenarios were used to change the value of health insurance status: (i) assigning all individuals in the sample as 'uninsured,' (ii) expansion of *Askes *insurance to all individuals in the sample, and (iii) expansion of *Jamsostek *to all individuals in the sample. For each scenario, a prediction was then made for each income level. The constant differences in the probabilities predicted under these scenarios (uninsured, *Askes*, and *Jamsostek*), therefore, are exclusively owing to the effects of insurance. Table 4 summarizes the results of the predictions.

**Table 4:** Predicted probabilities of provider usage under different insurance schemes and income quintiles

	**Unconditional estimates (%)**			**Conditional estimates (%)**		
		
	*Uninsured*	*Askes*	*Jamsostek*	*Uninsured*	*Askes*	*Jamsostek*
**Self-treatments:**						
Quintile 1^st ^(lowest)	84.77	77.20	74.74	75.67	65.44	62.45
Quintile 2^nd^	83.34	75.68	71.69	73.41	63.24	58.20
Quintile 3^rd^	81.80	74.14	68.88	71.27	61.29	54.83
Quintile 4^th^	81.22	73.84	66.88	70.99	61.77	53.16
Quintile 5^th^(highest)	79.55	73.11	62.78	68.51	60.63	48.06
Average	82.02	74.70	68.73	71.68	62.29	54.77
Ratio (Q-5^th^/Q-1^st^)	0.94	0.95	0.84	0.91	0.93	0.77

**Public providers:**						
Quintile 1^st ^(lowest)	12.34	20.01	16.50	19.69	30.32	24.65
Quintile 2^nd^	12.55	20.34	16.29	19.78	30.47	23.74
Quintile 3^rd^	12.81	20.66	16.09	19.87	30.55	22.97
Quintile 4^th^	12.05	19.60	14.80	17.94	27.87	20.01
Quintile 5^th^(highest)	10.23	16.74	11.79	14.85	23.35	15.36
Average	11.95	19.40	14.99	18.23	28.23	20.97
Ratio (Q-5^th^/Q-1^st^)	0.83	0.84	0.71	0.75	0.77	0.62

**Private providers:**						
Quintile 1^st ^(lowest)	2.90	2.79	8.75	4.63	4.24	12.90
Quintile 2^nd^	4.11	3.98	12.02	6.81	6.28	18.05
Quintile 3^rd^	5.39	5.20	15.03	8.86	8.16	22.20
Quintile 4^th^	6.73	6.56	18.32	11.07	10.35	26.83
Quintile 5^th^(highest)	10.22	10.14	25.43	16.64	16.02	36.58
Average	6.03	5.90	16.28	10.09	9.49	24.26
Ratio (Q-5^th^/Q-1^st^)	3.53	3.63	2.91	3.59	3.78	2.84

The first panel of Table 4 shows that about 72% of the uninsured who reported being ill opted, on average, for self-treatments compared with 62% for *Askes *beneficiaries and only 55% for *Jamsostek *members, suggesting that uninsured persons have the highest probability of using self-treatment. Individuals covered by *Askes *significantly demonstrated the highest probability of choosing public providers, consistent across all income quintiles (second panel). Evidence from the conditional estimates indicates that beneficiaries of *Askes *had, on average, a 55% higher probability (increasing from 18.2% to 28.2%) to use public providers than the uninsured. *Jamsostek *beneficiaries also had a 25% higher predicted probability to use outpatient care in public providers compared to the uninsured.

Table 4 also shows that the gap between the lowest- and highest-income quintiles of uninsured healthcare users was wider in private providers than public ones. The ratio of the highest to the lowest-income quintile among the uninsured, derived from a conditional estimation, was 0.75 (14.85/19.69) for public providers and 3.59 for private ones. The gap between the lowest and highest-income quintiles in private outpatient use among *Jamsostek *member was the smallest (2.9 and 2.8 de rived from unconditional and conditional estimates, respectively). It is also worth noting that the highest income bracket of uninsured people had the lowest probability of choosing self-treatment and the highest probability of using private providers.

**Figure 2:**
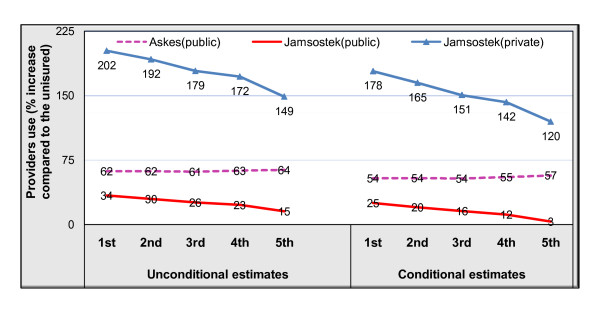
**The effects of health insurance on the use of public and private providers**. (Dash purple-line indicates the effects of *Askes *on the demand public outpatient care. Red-line and blue-line with triangle marker point to the effects of *Jamsostek *on the demand public and private outpatient care, respectively. In all lines, the value of the percentage (%) reveals the magnitude effects of health insurance on healthcare demand as compared to the uninsured).

Figure 2 depicts the effects of health insurance programs on the demand for public and private outpatient care. The greatest effect of *Jamsostek *insurance on both public and private outpatient use was found in the lowest income quintile. The effect declines as the quintile level increases. This pattern corresponds with the estimated coefficient of the interaction term between *Jamsostek *and income, which is always negative (see Table 3).

## Discussion

Estimating healthcare demand conditional on an event of illness poses a problem due to possibility endogeneity of self-reported illnesses resulting from sample selection bias [5,6,21]. Sample selection bias refers to the problem where the dependent variable is only observed for a restricted (non-random) sample. This study, however, confirms that conditional estimates do not suffer from the sample selectivity problem, in-line with a study conducted in Côte d'Ivoire [6]. Another problem with conditional estimates relates to the underreporting of incidents of illness in surveys [7]. However, this study minimizes the risk of underreporting by adopting two health status measurements (i.e., activity of daily living impairments and the incidence of severe illness) to capture the event of illness.

This study found that both unconditional and conditional estimates yielded similar results, especially in term of the sign of the variable of interest as well as most of the other covariates. However, the results suggest that conditional estimates yield a lower insurance effect on the utilization of outpatient care than unconditional ones. The effects of *Askes *on the use of public outpatient care were about 7.5 percent lower in the conditional estimates (55%) than in the unconditional ones (62%). The demand effects of *Jamsostek *for outpatient care with private providers were about 20 percent lower in the conditional estimates than in the unconditional ones (156% and 176%, respectively). This is inconsistent with the finding of a previous study. Dow found that conditional estimates yielded price elasticity about 25% higher than those derived from unconditional estimates [6]. Unconditional estimates are preferred since conditional estimates may be statistically biased. Even when properly estimated, such estimates can only be interpreted as short-run effects.

A critical question is when should we use unconditional estimates and when should we rely on conditional estimates? The answer depends on the purpose of the research. When the research aims to measure long-run price effects, unconditional estimates are the desired option. However, if the research is designed, for instance, to measure equity in healthcare utilization, conditional estimates are preferable [4,23]. Because conditional estimations do not suffer from statistical selection bias, they are acceptable for short-term analysis, and may even be preferable since they are less costly to implement. For instance, questionnaires need only be administered to those who are sick. Conditional surveys are worthwhile, especially in developing countries like in Indonesia, since research resources (i.e., time, money, manpower, etc.) are usually inadequate.

This study also investigated the effects of health insurance on healthcare demand. The findings show that health insurance has a strongly positive impact on the demand for outpatient care in Indonesia. This supports theories of health insurance [24], and concurs with previously published studies conducted in other contexts [17-19,25,26].

The findings reveal problems for the uninsured and their predicted probability of using outpatient care with private providers, particularly those in the lowest income quintile. Examining the ratio of healthcare use among the highest to lowest-income quintiles among uninsured people, we see that the lowest income groups are less likely to use private outpatient services. This is due to increasingly expensive private health facilities. The poor are therefore more likely to opt for cheaper treatments for their illness, such as using outpatient public facility or self-treatment (i.e., buying drug from a pharmacy or simply not seeking care at all). The implication for equitable outcomes in this situation gives cause for concern.

However, once people are covered by insurance, particularly those in the lowest income groups, they utilize substantially more health services. This study demonstrated an over-proportional demand effect of insurance with the effects more pronounced in the lowest income groups. These findings implicitly indicate that low-income people have a higher price elasticity of demand, a finding that is consistent with empirical evidence elsewhere [1,19,25,26]. A study done by Pradhan et al. (2007) also found that the effect of the targeted price subsidy offered through the health card program was largest for the poorest quintile [27]. From a public health perspective, these findings are of substantial interest. It suggests that expanding health insurance in Indonesia, as is the current policy thrust, will have a stronger impact on increasing formal care usage rates among the poor. The introduction of a demand-side subsidy to insure the 76.4 million poor in Indonesia is supported by the findings of this study.

Research findings also indicate that among uninsured people the poorest have a higher probability of using public providers than the richest quintile. Arguably, this is particularly the case with regards to the extensive subsidization of medical care costs by the government that keep user costs in public health facilities generally low. Mean spending on outpatient medical care was only 1.5% and 4.8% of total income for public and private health facilities, respectively. Therefore, poorest uninsured people who devoted on average about 4% of their income on healthcare are still able to afford healthcare. A study conducted in Indonesia also found that the share of household expenditures spent on health in 1997 was only 1.9% for urban areas and 1.6% for rural areas [10].

## Conclusion

This study estimates the effects of health insurance on healthcare demand in Indonesia using samples that are both unconditional and conditional on being ill. The latter approach does not suffer from the sample selectivity problem. Both estimations yield very similar outputs with respect to the direction of most of the covariates. The magnitude effects of insurance on demand for healthcare, however, are higher in the former estimates than the latter. The choice between using unconditional or conditional estimates for future studies should be determined by the main purpose of the research.

This study supports growing literature that health care demand is regressive irrespective of insurance status. Health insurance significantly improves access to health care services, with the largest demand effect of insurance found among individuals in the lowest income quintile. This study therefore supports the expansion of insurance programs or the establishment of a national health insurance program in order to address under-utilization of formal healthcare in Indonesia. A demand-side subsidy to pay insurance premiums for the poor is also recommended.

## Competing interests

The author declares that they have no competing interests.

## Authors' contributions

The author is fully responsible for all parts of the study. The author has made contributions to conception, design, managing data, running model and interpretation of results; has drafted the manuscript and has revised it critically for important intellectual content; and has approved the final version to be published.
